# A case report of Japanese encephalitis in Paracelis, Mountain Province, the Philippines

**DOI:** 10.5365/wpsar.2024.15.2.1049

**Published:** 2024-06-27

**Authors:** Fe S Mapangdol, Ray Justin C Ventura, Mariz Zheila C Blanco, Sheryl Racelis-Andrada, Rosario P Pamintuan, Rio L Magpantay, Karen B Lonogan

**Affiliations:** a Field Epidemiology Training Program – Intermediate Course; Luis Hora Memorial Regional Hospital, Abatan, Bauko, Mountain Province, Philippines.; b Department of Health, Manila, Philippines.; c Mariano Marcos Memorial Hospital and Medical Center, Batac, Ilocos Norte, Philippines.; d Department of Health Center for Health Development, Cordillera Administrative Region, Philippines.

## Abstract

On 12 September 2022, a 10-year-old female in Paracelis municipality, Mountain Province, the Philippines, without travel history outside the municipality, experienced acute onset of fever and a change in mental status with disorientation, an altered level of consciousness and new onset of seizures. She was hospitalized at the district hospital from 1 to 3 October 2022, before being transferred to the regional hospital. As diphtheria was originally suspected, the investigation team reviewed records and reports and interviewed key informants to gather additional information and organize case finding and contact tracing. The patient’s condition was laboratory-confirmed for Japanese encephalitis virus infection. An environmental survey was carried out at the patient's residence to check for the presence of vectors and contributing factors. Exemplifying inadequate vaccination coverage for Japanese encephalitis virus in Mountain Province, the patient had not been vaccinated against the disease. It is recommended that vaccination campaigns be immediately implemented in the affected area and the surveillance system be strengthened for early detection and prompt response to the emergence of cases and outbreaks. Overall, the investigation highlighted the importance of strong surveillance and response systems for early detection and control of diseases, such as Japanese encephalitis virus. It also underscores the need for comprehensive vaccination programmes to prevent outbreaks and protect vulnerable populations.

On 3 October 2022, a suspected case of diphtheria from the district hospital in Paracelis municipality was referred to the regional hospital in Mountain Province, the Philippines. An event-based surveillance and response report was forwarded to the Provincial Health Office of Mountain Province. On 10 October, a team from the Philippines Field Epidemiology Training Program (FETP) began an investigation, during which laboratory testing confirmed that the patient was infected with Japanese encephalitis virus (JEV).

Paracelis is one of 10 municipalities of Mountain Province, situated within the Cordillera Administrative Region in Luzon, the largest and northernmost island group of the Philippines. Paracelis is a border town of Mountain Province, sharing borders with the provinces of Kalinga, Isabela and Ifugao. The municipality has a land area of 570 km^2^, with nine barangays (villages). According to the Philippine Statistics Authority, as of 2020, Paracelis had a population of 31 168. (*1*) The population’s main livelihood activity is farming. Paracelis has one district hospital with a 25-bed capacity, one Rural Health Unit and nine barangay health stations.

The first recorded case of Japanese encephalitis (JE) was in the 1870s in Japan. (*2*) Since then, the disease has been found across Asia and has become the most common cause of epidemic encephalitis globally. (*2*) JEV has four currently recognized genotypes, but the origin of the virus remains unknown. The JE serogroup belongs to the genus *Flavivirus*, family *Flaviviridae*. The virus is found in pigs and birds and is transmitted by mosquitoes, principally by *Culex tritaeniorhynchus* when they bite infected animals, which then act as vectors to end hosts such as humans. (*2*, *3*) Transmission occurs most commonly in agricultural areas such as farms and rice paddies but may also occur in urban areas where flooding irrigation attracts wading birds. (*4*) The incubation period averages 6–8 days but can range from 4 to 15 days. The most common signs and symptoms are fever, nausea, vomiting, diarrhoea and myalgia, which may last for several days. Neurological manifestations may include altered mental status, agitation, confusion, psychosis, headache, seizure and flaccid paralysis. (*4*)

The global incidence of JE is unknown, but the World Health Organization (WHO) estimates  that there are approximately 68 000 clinical cases and approximately 13 600–20 400 deaths from the disease per year globally. (*5*) The Philippines is endemic for JEV, with cases recorded in every region in the country. JEV is the cause of 15% of all acute encephalitis cases in the Philippines. (*6*) According to WHO data, 988 JE cases were recorded in 2021 and 1532 in 2020. (*7*) The incidence in the Philippines is around 0.7/100 000 in children aged < 15 years, with the incidence higher in the northern region of the country. (*8*) In Mountain Province, cases have been reported each year since 2015 from the municipalities of Natonin and Paracelis.

In 2019, a JE vaccination programme was undertaken for children aged 9–59 months in the northern regions of the Philippines – Regions 1, 2 and 3, and the Cordillera Administrative Region. The Mountain Province was selected for this supplemental immunization activity within the Cordillera Administrative Region, with the Paracelis municipality among the 10 provinces of Mountain Province.

## CASE REPORT

The case investigation objectives were to: (1) confirm the diagnosis; (2) profile the case; (3) identify the source and mode of transmission; and (4) recommend control and prevention measures.

Informed consent was obtained from the patient’s parents at the start of the investigation. As part of the FETP team’s investigation, the medical records of the patient at the district and regional hospitals were reviewed to establish a timeline of events. The 10-year-old female patient had been residing in Paracelis since 2020 and had reportedly been fully immunized. However, no vaccination records were found in her medical records or at the Municipal Health Office. The patient would not have been vaccinated against JE during the vaccination roll-out in 2019 since she was not in the target age group of 9–59 months at that time.

### Timeline of events

During the second week of September 2022, the patient reported pain during swallowing, which was relieved by drinking vinegar. On 29 September, the patient reported a febrile episode accompanied by headache and poor oral intake. Her caregiver opted for home management. It was noted that 1–2 years before this episode, the patient had experienced recurring boils on her head and had constantly reported abdominal pain. However, no medical consultation was made then; rather, traditional healing methods were used, which included applying a chewed betel nut on the affected area.

On 1 October 2022, due to persistent fever and headache, accompanied by abdominal pain, malaise, dizziness and poor oral intake, the caregiver took the patient to the district hospital. During admission, the patient had bouts of vomiting, was non-conversant, showed decreased response to stimuli, and was ambulatory but needed assistance. Upon assessment, she had dry mouth, enophthalmos, and was febrile. No inspection of the patient’s oral mucosa was made. In the ward, the patient was observed to have weakness in her lower extremities and needed full-time assistance from the caregiver for daily activities.

On the afternoon of 2 October, the patient experienced a sudden decrease in sensorium with a Glasgow coma scale assessment of 5/15. A referral was made to the attending paediatrician, who recommended transferring the patient to a facility that could provide a higher level of care.

While being transported to the hospital emergency department, the patient had an episode of seizure and decorticate posturing of the extremities. On admission, the patient was unresponsive, with fixed dilated pupils and a Glasgow coma scale assessment of 6/15. The patient was intubated, during which a whitish biofilm on her tonsillar area was noted. She was admitted for 41 days and was discharged from the facility on 13 November, with a Glasgow coma scale score of 6/15. The patient had spontaneous eye opening, no motor reflexes, and needed a full-time caregiver.

### Laboratory confirmation

On 5 October, throat swab and serum samples were collected from the patient for diphtheria and JEV confirmatory tests, respectively. Stool specimens for an acute flaccid paralysis (AFP) confirmatory test were collected on 15 and 16 October and sent to the Research Institute for Tropical Medicine. A cerebrospinal fluid sample was not collected, as performing a lumbar tap was deemed by her physicians to be detrimental to the patient.

On 12 October, a negative *Corynebacterium diphtheriae* isolate was received and on 3 November, a negative result for AFP was received. Finally, on 4 November, JEV infection was confirmed by the presence of JEV-specific IgM in the serum sample.

## FIELD INVESTIGATION

### Review of records

A review of records at the Rural Health Unit and the Provincial Health Office was conducted to determine the JE vaccination coverage within the municipality and in Mountain Province, respectively.

### Key informant interview

A face-to-face interview was conducted with a municipal health officer at the Rural Health Unit and a nurse at the district hospital. The municipal health officer reported that from 2015 to 2022 there had been two additional confirmed JE cases in the municipality. The nurse reported that no JE cases had been seen at the hospital except for the current case. The timeline of events reported by the patient was validated during these interviews.

### Active case finding

To determine if there were other cases, the patient’s siblings were interviewed together with their aunt, and the patient’s teacher and classmates. Informed consent was obtained from all adult interviewees and the parents or guardians of all child interviewees. A standard questionnaire was used to determine if these individuals had the same signs and symptoms presented by the patient that met the case definition. A suspected case was defined as a previously well individual residing in Paracelis, Mountain Province, who had an acute onset of fever and a change in mental status (including symptoms such as confusion, coma or an inability to talk) and/or new onset of seizures (excluding simple febrile seizures) from 24 September to 1 October 2022. A confirmed case was defined as a suspected case with JEV-specific IgM antibody present in a blood sample. No additional cases were found during active case finding.

### Vaccine coverage and other cases

Among the 10 municipalities of Mountain Province, the coverage of the JE vaccination programme in 2019 for children aged 9–59 months ranged from 87% to 99%. Paracelis had one of the lowest vaccination rates at 88% (**Table 1**).

**Table 1:** Japanese encephalitis vaccination coverage by municipality among children aged 9–59 months, Mountain Province, Philippines, 2019

Municipality	No. eligible for vaccine^a^	No. of children immunized^b^			Vaccination coverage (%)	Vaccination target	
		9–11 months	12–59 months	Total		No.	%
**Besao**	**620**	**21**	**453**	**474**	**76.5**	**475**	**99.8**
**Natonin**	**904**	**25**	**667**	**692**	**76.6**	**703**	**98.4**
**Sagada**	**979**	**25**	**822**	**847**	**86.5**	**861**	**98.4**
**Sabangan**	**820**	**31**	**659**	**690**	**84.2**	**715**	**96.5**
**Tadian**	**1706**	**66**	**1132**	**1198**	**70.2**	**1253**	**95.6**
**Bauko**	**2734**	**76**	**2067**	**2143**	**78.4**	**2329**	**92.0**
**Bontoc**	**2169**	**67**	**1292**	**1359**	**62.7**	**1490**	**91.2**
**Barlig**	**424**	**23**	**198**	**221**	**52.1**	**247**	**89.5**
**Paracelis**	**2475**	**97**	**2271**	**2368**	**95.7**	**2686**	**88.2**
**Sadanga**	**774**	**16**	**462**	**478**	**61.8**	**552**	**86.6**

^a^ Based on the projected population from the Philippine Statistics Authority.

^b^ Based on the number of children immunized compiled by health-care workers.

There were six laboratory-confirmed cases of JE in Mountain Province between 2015 and 2022, of which three were in Paracelis municipality and the other four were in Natonin municipality.

### Environmental investigation

A visual survey was conducted at the patient's residence to check for the presence of vectors and any contributing factors regarding other diseases being considered. A larval survey was conducted in two villages in Paracelis municipality on 17 November, during which 44 larvae and 23 pupae were collected from 323 containers in 100 households.

During the visual survey at the case’s residence, a pig was observed inside the house, along with carabaos and ducks nearby. Natural and artificial breeding sites were also observed around the house.

The larval survey found that most of the larvae (27/44, 61.4%) and pupae (13/23, 56.5%) that were identified were *Aedes albopictus*. The second most abundant species was *Aedes aegypti* (13/44, 29.5%). Small numbers of *Culex* larvae and pupae were also found (4/44, 9.1% and 1/23, 4.3%, respectively) (**Table 2**).

**Table 2:** Results of the larval survey conducted in Paracelis, Mountain Province, 17 November 2022

Mosquito species	Larvae		Pupae	
	No.	%	No.	%
***Aedes albopictus***	**27**	**61.4**	**13**	**56.5**
***Aedes aegypti***	**13**	**29.5**	**9**	**39.1**
***Culex***	**4**	**9.1**	**1**	**4.3**
**Total**	**44**	**–**	**23**	**–**

## Discussion

This is a report of a case of laboratory-confirmed JE in a 10-year-old female from Paracelis, Mountain Province, the Philippines. The signs and symptoms presented by the patient were strong indications of this disease, which was further confirmed by laboratory testing of a serum specimen for the presence of JEV-specific IgM.

The field investigation was able to identify a plausible cause of transmission, which was directly related to the natural and artificial breeding sites around the house. *Culex* and *Aedes albopictus* larvae and pupae were detected in the environmental investigation, and these are both competent vectors to transmit JEV. (*9*) The fact that the investigation did not include the identification of *Culex tritaeniorhynchus* is a limitation of the study. However, the presence of a pig inside the case’s residence strengthened the evidence for the diagnosis of JE, as pigs are its natural amplifying host, while mosquitoes are the vectors to both animals and humans, who are dead-end hosts for JEV. (*10*)

Vaccination has dramatically reduced the number of JE cases. A study conducted in Yunnan province, China, showed a decrease in incidence rate per 100 000 population, from 1.16 in 2009 to 0.17 in 2017, with the introduction of a JE vaccination programme. (*11*) However, as the virus is maintained in animal reservoirs, non-immune individuals remain at risk of infection. In the Philippines, the JE vaccine is not widely available to the public and is only available through private clinics. Additionally, the case was not in the age group that was covered during the 2019 vaccination programme in Mountain Province.

JE usually affects children with low socioeconomic status. In one study conducted in a hospital in India, the age group that was predominantly affected by JE was 5–12 years. Most cases were from rural areas belonging to a low socioeconomic group, where most of the children were unvaccinated. (*12*) The present case belongs to the same age group.

The case manifested weakness in lower limbs earlier on, which fits the case definition of AFP and may be attributed to JE. One report from West China Hospital of Sichuan University described a case with an initial manifestation of AFP on the right upper limb, who was later confirmed to have JE. (*13*) In another report from Indonesia, a 29-year-old female also developed flaccid paralysis and was later laboratory-confirmed to have JE.

During the investigation, the team recommended the immediate implementation of JE vaccination campaigns in the affected area, strengthening of the surveillance system for early detection, prompt response to outbreaks, and for the local government to implement and sustain strategies to reduce mosquito breeding sites and mosquito avoidance measures. The investigation team also recommended that the municipal health office conduct a community-wide assembly to encourage the observance of “Oplan Taob,” a campaign to encourage reducing mosquito breeding sites around the community, wearing long-sleeved clothes and using mosquito repellents. This campaign is part of the 4S Strategy (Search and destroy mosquito breeding sites, Self-protection, Seek early consultation, Support fogging in case of an outbreak). (*14*) These measures would help to reduce the risk of not only JE, but also dengue and other mosquito-borne diseases endemic to the area.

Overall, this investigation underscored the need for a comprehensive vaccination programme to prevent outbreaks and protect vulnerable populations, and the need for further training among health-care workers on the early detection of JE cases for prompt management. Further training of sanitary inspectors and other public health personnel on mosquito capture and identification is also recommended to improve vector control efforts.
